# Involvement of PACLOBUTRAZOL RESISTANCE6/KIDARI, an Atypical bHLH Transcription Factor, in Auxin Responses in Arabidopsis

**DOI:** 10.3389/fpls.2017.01813

**Published:** 2017-10-24

**Authors:** Kaijie Zheng, Yating Wang, Na Zhang, Qiming Jia, Xutong Wang, Chunjiang Hou, Jin-Gui Chen, Shucai Wang

**Affiliations:** ^1^Key Laboratory of Molecular Epigenetics of MOE, Institute of Genetics and Cytology, Northeast Normal University, Changchun, China; ^2^Key Laboratory of Soybean Molecular Design Breeding, Northeast Institute of Geography and Agroecology, Chinese Academy of Sciences, Changchun, China; ^3^Biosciences Division, Oak Ridge National Laboratory, Oak Ridge, TN, United States

**Keywords:** ARF5, ARF8, Arabidopsis, auxin, PRE6, transcription factor

## Abstract

Auxin regulates nearly all aspects of plant growth and development including cell division, cell elongation and cell differentiation, which are achieved largely by rapid regulation of auxin response genes. However, the functions of a large number of auxin response genes remain uncharacterized. Paclobutrazol Resistance (PRE) proteins are non-DNA binding basic helix-loop-helix transcription factors that have been shown to be involved in gibberellin and brassinosteroid signaling, and light responses in Arabidopsis. Here, we provide molecular and genetic evidence that *PRE6*, one of the six *PRE* genes in Arabidopsis, is an auxin response gene, and that PRE6 is involved in the regulation of auxin signaling. By using quantitative RT-PCR, we showed that the expression level of *PRE6* was increased in response to exogenously applied IAA. GUS staining results also showed that the expression of *GUS* reporter gene in the *PRE6p:GUS* transgenic seedlings was elevated in response to auxin. Phenotypic analysis showed that overexpression of *PRE6* in Arabidopsis resulted in auxin-related phenotypes including elongated hypocotyl and primary roots, and reduced number of lateral roots when compared with the Col wild type seedlings, whereas opposite phenotypes were observed in the *pre6* mutants. Further analysis showed that *PRE6* overexpression plants were hyposensitive, whereas *pre6* mutants were hypersensitive to auxin in root and hypocotyl elongation and lateral root formation assays. By using protoplasts transfection, we showed that PRE6 functions as a transcriptional repressor. Consistent with this, the expression of the auxin response reporter *DR5:GUS* was decreased in *PRE6* overexpression lines, but increased in *pre6* mutants. When co-transfected into protoplasts, ARF5 and ARF8 activated the expression of the *PRE6p:GUS* reporter. Chromatin immunoprecipitation assays showed that ARF5 and ARF8 can be recruited to the promoter regions of *PRE6*. Taken together, these results suggest that *PRE6* is an auxin response gene whose expression is directly regulated by ARF5 and ARF8, and that PRE6 is a transcriptional repressor that negatively regulates auxin responses in Arabidopsis.

## Introduction

Auxin regulates many aspects of plant growth and development such as cell division, cell elongation and cell differentiation, mainly via rapid regulation of auxin response genes (Teale et al., 2006). The components involved in auxin signaling and the mechanisms of auxin perception and signal transduction have been largely elucidated during the last several decades (Chandler, 2016; Weijers and Wagner, 2016; Mironova et al., 2017). The core components involved in auxin signaling pathway include the TIR1/AFB (TRANSPORT INHIBITOR RESISTANT 1/AUXIN SIGNALING F-BOX) proteins, the ARF (AUXIN RESPONSE FACTOR) transcription factors, and the Aux/IAA (AUXIN/INDOLE-3-ACETIC ACID) proteins (Weijers and Wagner, 2016). The TIR1/AFB proteins are auxin receptors that can be activated by auxin molecules (Dharmasiri et al., 2005a,b; Kepinski and Leyser, 2005; Parry et al., 2009), the ARF transcription factors can bind to the TGTCTC auxin response elements in the promoter regions of the auxin response genes (Ulmasov et al., 1997a, 1999; Tiwari et al., 2003; Wang et al., 2005; Guilfoyle and Hagen, 2007; Chandler, 2016), whereas the Aux/IAA proteins are transcriptional repressors that can interact with ARF transcription factors (Tiwari et al., 2003, 2004).

In Arabidopsis, five ARFs including ARF5, ARF6, ARF7, ARF8, and ARF19 have been shown to be transcriptional activators (Wang et al., 2005; Guilfoyle and Hagen, 2007). When auxin levels in the cells are low, the Aux/IAA proteins are stable, and they can interact with the ARF activators that bound to the auxin response elements, thus repressing the activities of the ARF activators, resulting in repression of the auxin response genes (Li et al., 2011; Vernoux et al., 2011; Farcot et al., 2015). Elevated auxin levels in the cells will result in the activation of the TIR1 auxin receptor, leading to the ubiquitylation of the Aux/IAA proteins. Ubiquitinated Aux/IAA proteins will be degraded, allowing the activation of the auxin response genes by the ARF activators (Dharmasiri et al., 2005a,b; Kepinski and Leyser, 2005; Guilfoyle and Hagen, 2007; Mockaitis and Estelle, 2008). Although many auxin response genes have been reported, many others remain unidentified.

The bHLH (basic helix-loop-helix) transcription factor family is one of the largest transcription factor families in Arabidopsis, which regulates multiple aspects of plant growth and development (Bailey et al., 2003; Zhao et al., 2012). Among the bHLH genes in Arabidopsis, a total of six genes encode Paclobutrazol Resistances (PREs), which are atypical bHLH transcription factors that lack the basic domain required for DNA binding (Lee et al., 2006; Mara et al., 2010). Accumulated evidence suggest that PREs are involved in the regulation of plant growth and development as well as response to plant hormones and environmental stimuli such as temperature and light (Lee et al., 2006; Wang et al., 2009; Zhang et al., 2009; Mara et al., 2010; Bai et al., 2012; Castelain et al., 2012; Hao et al., 2012; Ikeda et al., 2012, 2013).

PRE1/BANQUO1(BNQ1)/BHLH136 is one of the first identified PRE transcription factors. It was identified by screening Arabidopsis activation-tagged transgenic lines with enhanced resistance to Paclobutrazol (Lee et al., 2006). Overexpression of *PRE1* resulted in gibberellin related phenotypes such as longer hypocotyl, elongated petioles and early flowering (Lee et al., 2006). Overexpression of *PRE2/BNQ2/BHLH134*, *PRE3/ATBS1/TMO7*, *PRE4/BNQ3/BHLH161* and *PRE5* also resulted in a phenotype similar to that of the *PRE1* transgenic plants (Lee et al., 2006), while the *kidari/pre6-D* mutant plants showed less rosette leaves and conditional longer hypocotyl under blue light (Hyun and Lee, 2006). Consistent with these observation, *PRE1-RNAi*, *PRE2-RNAi* and *pre3* mutant plants flowered later than the Col wild type plants (Mara et al., 2010), suppressing the expression of *PRE2*, *PRE5*, and *PRE6/KIDARI* by artificial microRNA resulted in dwarfed phenotype and decreased sensitivity to gibberellin (Hyun and Lee, 2006; Bai et al., 2012; Oh et al., 2014), and knock down mutants of *PRE1*, *PRE2*, *PRE5*, and *PRE6* by artificial micro-RNA resulted in smaller leaves and shorter hypocotyl in the transgenic plants (Bai et al., 2012; Oh et al., 2014).

PREs have been also shown to be involved in the regulation of brassinosteroid and light signaling. PRE6/KIDARI(KDR) was originally identified through a genetic screen of activation tagged mutants for long hypocotyl phenotype under blue and far-red light conditions (Hyun and Lee, 2006). PRE3 is able to suppress the phenotype of brassinosteroid receptor mutant *bri1*, and is involved in the regulation of light signaling (Wang et al., 2009; Castelain et al., 2012). PRE1 also plays a role in the regulation of brassinosteroid and light signaling (Zhang et al., 2009; Mara et al., 2010). On the other hand, the *pre4* mutants have light signaling related phenotypes including pale-green sepals and carpels, decreased chlorophyll levels and late flowering (Mara et al., 2010), and suppression of *PRE2*, *PRE5*, and *PRE6* led to decreased sensitivity to brassinosteroid, but increased sensitivity to light signaling (Hyun and Lee, 2006; Bai et al., 2012; Oh et al., 2014).

In addition to gibberellin and brassinosteroid related phenotypes, *PRE3* overexpression plants showed auxin related phenotypes including longer primary root and decreased lateral root density, which can be restored by exogenously applied IAA treatment (Castelain et al., 2012). PRE6 has recently been reported to be involved in shade avoidance response, a phenomenon related to auxin signaling (Tian and Reed, 2001; Halliday et al., 2009; Sassi et al., 2013; Gommers et al., 2017). Most importantly, PRE3 has been identified as a target of ARF5/MONOPTEROS (MP) (Schlereth et al., 2010), and PRE1 has been reported to cooperate with ARF6 to regulate cell elongation in Arabidopsis (Oh et al., 2014), suggesting that PREs may be involved in the regulation of auxin signaling.

In this study, we report the identification of *PRE6* as an auxin response gene, we show that *PRE6* is a target of ARF5 and ARF8, and we provide evidence that PRE6 is a transcriptional repressor and it negatively regulates auxin responses in Arabidopsis.

## Materials and Methods

### Plant Materials and Growth Conditions

All the mutants and transgenic plants are in the Columbia-0 (Col) background, and Col Arabidopsis was used for plant transformation and protoplast isolation. The T-DNA insertion lines for *PRE6/KDR* (At1g26945), SALK_033495C, and SALK_048383C were obtained from the ABRC (Gommers et al., 2017), and designated as *pre6-1* and *pre6-2*, respectively. The *DR5:GUS* transgenic plants have been described previously (Ulmasov et al., 1997b). The *DR5:GUS*/*pre6* and *DR5:GUS*/*35S:PRE6* plants were generated by crossing *DR5:GUS* transgenic plants with *pre6-1* mutant or *35S:PRE6-1* transgenic plants, and selecting lines that were homozygous for both the *DR5:GUS* reporter gene and the *pre6* mutant or the *35S:PRE6* transgenic plant, respectively.

For protoplast isolation and plant transformation, Col wild type Arabidopsis seeds were germinated and grown in soil pots as described previously (Wang et al., 2015).

For RNA isolation, Arabidopsis seeds were surface-sterilized and sown on 0.6% (w/v) phytoagar (PlantMedia) solidified 1/2 MS (Murashige and Skoog) medium with vitamins (PlantMedia) and 1% w/v sucrose as described previously (Wang et al., 2015; Dai et al., 2016).

For phenotypic and auxin response analyses in seedlings, surface-sterilized seeds were sown and grown on 1.5% (w/v) phytoagar solidified 1/2 MS medium.

All plants were grown in a growth chamber at 22°C, with a 16 h/8 h photoperiod and a photon density at ∼125 μmol m^-2^ s^-1^, or under darkness in the case of hypocotyl length analysis.

### RNA Isolation, RT-PCR, and Quantitative RT-PCR (qRT-PCR)

To examine the expression of *PREs* and *Aux/IAA* genes in response to auxin, 14-day-old Col wild type, *pre6* mutants and *35S:PRE6* transgenic seedlings were transferred into plates containing 10 μM IAA and kept in darkness for 4 h on a shaker, then total RNA was isolated using EasyPure Plant RNA Kit (TransGen Biotech) by following the manufacturer’s instructions. To examine the expression of *PRE6* in the *pre6* mutants and the *35S:PRE6* transgenic plants, total RNA was isolated from 7-day-old seedlings.

Two μg of total RNA was subjected to cDNA synthesis via Oligo(dT)-primed reverse transcription by using the EasyScript First-Strand cDNA Synthesis Super Mix (TransGene Biotech). Synthesized cDNA was used for PCR reactions, and expression of *ACTIN2* (*ACT2*) was used as a control.

The primers used for qRT-PCR analysis of *Aux/IAA* genes have been described previously (Liu et al., 2015). The *ACT2* primers used for qRT-PCR and the *ACT7* primers used for ChIP assay have been described previously (Wang et al., 2015). All other primers used in this study for PCR, RT-PCR, and qRT-PCR are listed in **Table 1**.

**Table 1:** Primers used in this study.

Primers	Sequences
*PRE1-NdeIF*	CAACATATGTCGAACAGAAGATCAAGG
*PRE1-SacIR*	CAAGAGCTCTTACATGAGTAGGCTTCTAATAACG
*PRE2-NdeIF*	CAACATATGTCTTCTAGCAGAAGGTCG
*PRE2-SacIR*	CAAGAGCTCTTATCCATTAATCAAGCTCCTAATAAC
*PRE3-NdeIF*	CAACATATGTCGGGAAGAAGATCAC
*PRE3-AflIIR*	CAACTTAAGTTATTGGGTAAGTAAGCTTCTG
*PRE4-NdeIF*	CAACATATGTCTAGCAGAAAATCACGTTC
*PRE4-SacIR*	CAAGAGCTCCTACTGCATAAGCAAACTTCG
*PRE5-NdeIF*	CAACATATGTCTAACAGAAGATCAAGACAAAC
*PRE5-SacIR*	CAAGAGCTCTTACATGAGTAAGCTTCTAATCACG
*PRE6-NdeIF*	CAACATATGTCTAGCAGAAGATCATCACG
*PRE6-SacIR*	CAAGAGCTCTTAATAATTAAGCAAGCTCCTAATGATGG
*PRE6p-PstIF*	CAACTGCAGGTGGTTAGTGTAGAGTC
*PRE6p-SacIR*	CAAGAGCTCCTTCTTTCTTGATATATTATAAG
*P1*	ATTATAAGTGTGTTTGTTTGGGTGT
*P2*	TCAAGAAGTTGTTCTCGTGGGA
*P3*	AGTCCGTATAATGTGCAGAGTC
*P4*	ACGACTCGTATGAGACGATACA
*P5*	ACATCAAAGGTCAAACATGGATG
*P6*	GCCTATCTCTGCATCTACCACA


### Constructs

The effector constructs *GD*, *CAT*, *LD-VP*, *ARF5*, *ARF6*, *ARF7*, *ARF8* and *ARF19*, and the reporter construct *LexA-Gal4:GUS* used for protoplast transient transfection assays have been described previously (Tiwari et al., 2001, 2004; Wang et al., 2005, 2007).

To generate HA-, GFP- or GD-tagged PRE6 constructs for plant transformation and protoplast transfection, full-length open reading frame (ORF) of *PRE6* was amplified by RT-PCR using RNA isolated from Col wild type seedlings, and cloned in frame with an N-terminal HA, GFP or GD tag, respectively, into the *pUC19* vector under the control of the double *CaMV 35S* promoter (Tiwari et al., 2001; Wang et al., 2005). The *35S:HA-PRE6* (referred as *35S: PRE6*) and the *35S:GFP-PRE6* constructs in *pUC19* was digested with *NdeI* and *SacI* and subcloned into the binary vector *pPZP211* (Hajdukiewicz et al., 1994) for plant transformation.

To generate the *PRE6p:GUS* construct, a 3571bp DNA fragment immediately before the start codon of the *PRE6* gene was PCR amplified using DNA isolated from Col wild type seedlings, and used to replace the *OFP1* promoter in the *OFP1p:GUS* in *pUC19* (Wang et al., 2007). The *PRE6p:GUS* construct in *pUC19* was digested with *PstI* and *SacI* and subcloned into the binary vector *pPZP211* for plant transformation.

### Plant Transformation and Transgenic Plants Selection

About 5-week-old Col wild type plants with several mature flowers were transformed with the *35S:PRE6*, *35S:GFP-PRE6* and *PRE6p:GUS* constructs in *pPZP211*, respectively, via *Agrobacterium tumefaciens* strain GV3101 by using the floral dip method (Clough and Bent, 1998). T1 transgenic plant seedlings were selected on 1/2 MS plates containing 50 μg/L kanamycin and 100 μg/L carbenicillin. A minimum of 15 independent transgenic lines with similar phenotypes were obtained for each construct, the expression status of the transformed genes was confirmed by RT-PCR or GUS staining, and two independent homozygous transgenic lines (T3 and/or T4) were used for further analysis.

### Auxin Sensitivity Assays

In the hypocotyl elongation assay, sterilized seeds of the Col wild type, *pre6* mutants and *35S:PRE6* transgenic lines were germinated and grown on vertically placed 1/2 MS plates supplied with different concentrations of IAA in a growth room under darkness. Photographs were taken from 5-day-old etiolated seedlings, and ImageJ software was used to measure the hypocotyl length.

In the primary root elongation and lateral root formation assays, 5-day-old seedlings of the Col wild type, *pre6* mutants and *35S:PRE6* transgenic lines grown on a vertically placed 1/2 MS plates were transferred to new 1/2 MS plates supplemented with different concentrations of IAA, and grown vertically for another 5 days. The positions of the primary root tips were marked immediately and 5 days after the transfer. The new elongated primary roots between the two marks were measured, and the number of lateral roots was counted after 5 days of the transfer.

In all the assays, 25–35 seedlings for each genotype were used, and student *t*-test^1^ was used for statistical analysis.

### Plasmid DNA Isolation, Protoplast Isolation and Transfection

Plasmid DNA of the reporter and effectors were isolated using the GoldHi Endo Free Plasmid Maxi Kit (CWBIO) by following the manufacturer’s protocol. Protoplasts were isolated from rosette leaves of 3–4-week-old Col wild type plants, and transfected as described previously (Tiwari et al., 2003; Wang et al., 2005, 2007, 2015; Tian et al., 2015; Dai et al., 2016; Zheng et al., 2016).

To examine the transcriptional activity of PRE6, plasmids of the reporter gene *LexA-Gal4:GUS* and the effector genes *LD-VP* and *GD-PRE6* or *GD* were co-transfected into protoplasts. To examine the activation of *PRE6* by ARF activators, plasmids of the reporter gene *PRE6p:GUS* and the *ARF* effector genes were co-transfected into protoplasts. To examine the association of ARF5 and ARF8 with the promoter region of *PRE6*, plasmids of *ARF5* or *ARF8* were transfected into protoplasts. Transfected protoplasts were incubated at room temperature in darkness for 20–22 h for GUS activity assays or ChIP assay.

### GUS Staining and GUS Activity Assays

Glucuronidase (GUS) activities in transfected protoplasts were measured using a Synergy^TM^ HT microplate reader.

To examine the auxin response of the *PRE6p:GUS* and effects of PRE6 on the expression of the *DR5:GUS* reporter gene, 7-day-old seedlings of *PRE6p:GUS*, *DR5:GUS*, *DR5:GUS/pre6* and *DR5:GUS/35S:PRE6* were treated with 10 μM IAA for 12 h, and then used for GUS activity assays either by staining or by quantitative measurement.

For GUS staining, Arabidopsis seedlings or different tissues or organs were incubated in solution containing *X*-Gluc (5-bromo-4-chloro-3-indolyl-β-D-glucuronide, Rose Scientific Ltd.) as described previously (Ulmasov et al., 1997b). For quantitative measurement, Arabidopsis seedlings were frozen in liquid nitrogen, then proteins were extracted and GUS activity was measured as described previously (Strader and Bartel, 2009).

### Chromatin Immunoprecipitation (ChIP) Assay

Chromatin immunoprecipitation (ChIP) assay was performed by using the procedure described previously (Wang et al., 2015) with some modification. Briefly, after incubation, the transfected protoplasts were cross-linked in 1% formaldehyde for 20 min, the chromatin complex was then sheared by sonication and immunoprecipitated by HA-antibodies or rabbit pre-immune serum as control, and the Chromatin–antibody complexes were collected by using protein *A*-agarose beads (Millipore). After washing, the DNA-protein cross-links obtained were reversed at 65°C for 12 h, and DNA was purified using PCR Cleanup Kit (Axygen) for PCR reactions.

### Microscopy

Photographs of the seedlings and the GUS stained tissues and organs were taken under a Motic K microscope equipped with an EOS 1100D digital camera. Photographs of the GFP fluorescence in the *35S:GFP-PRE6* transgenic seedlings were taken under an Olympus FV1000 confocal microscope.

## Results

### *PRE6* Is an Auxin Response Gene

**FIGURE 1:**
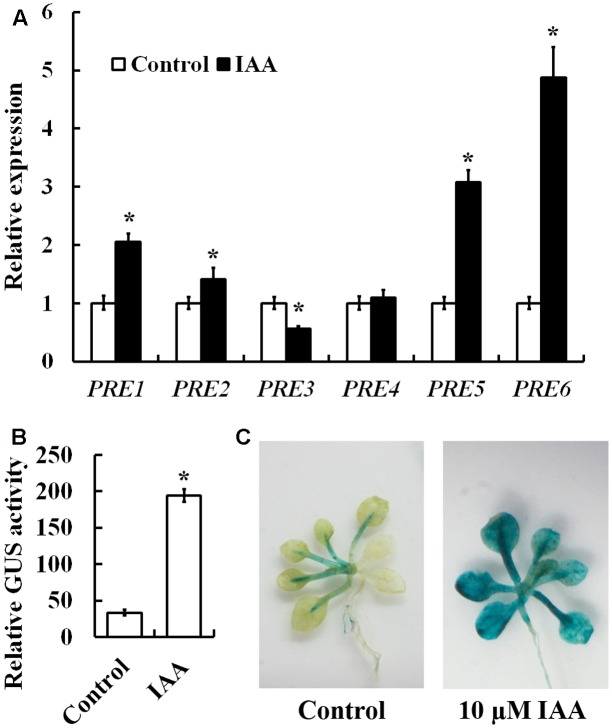
Induction of *PRE* genes by auxin and expression pattern of *PRE6*. **(A)** Expression of *PREs* in response to exogenously applied IAA. Fourteen-day-old seedlings were treated with 10 μM IAA for 4 h, then total RNA was isolated and subjected to quantitative RT-PCR analysis. *ACT2* was used as a reference gene, and expression of each *PRE* gene in the absence of IAA was set as 1. Data represent the mean ± SD of three replicates. ^∗^Significantly different from control (*p* < 0.01). **(B)** Auxin response of the *PRE6p:GUS* in transfected protoplasts. Plasmids of *PRE6p:GUS* were transfected into protoplasts isolated from rosette leaves of 3 to 4-week-old Col wild type plants, and GUS activity was measured after the protoplasts were incubated at room temperature in darkness for 20–22 h. Data represent the mean ± SD of three replicates. ^∗^Significantly different from control (*p* < 0.01). **(C)** Auxin response of the *PRE6p:GUS* reporter gene in the transgenic seedlings. Fourteen-day-old seedlings of the *PRE6p:GUS* transgenic plants were treated with 10 μM IAA for 4 h, then GUS activity was stained 12 h after treatment by using *X*-Gluc. Left, control seedlings, right, seedlings treated with IAA.

To examine whether PREs are involved in auxin signaling, we first examined the expression of *PRE* genes in response to auxin. Arabidopsis seedlings were treated with IAA, and qRT-PCR was used to examine the expression of *PRE* genes. As shown in **Figure 1A**, an ∼5-folds increase in response to auxin treatment was observed for *PRE6*, followed by ∼3, ∼2, and ∼1.5-folds for *PRE5*, *PRE1*, and *PRE2*, respectively. On the other hand, little increase was observed for *PRE4* (**Figure 1A**). Unexpectedly, we found that expression of *PRE3* was slightly suppressed by auxin treatment (**Figure 1A**).

PRE6 has been shown to be involved in light response and shade avoidance response (Hyun and Lee, 2006; Gommers et al., 2017). Because *PRE6* is the most strongly induced *PRE* gene by auxin (**Figure 1A**), we thus wanted to investigate the potential role of PRE6 in auxin signaling in Arabidopsis. We first made a *PRE6p:GUS* reporter construct and examined its expression in response to exogenously applied IAA in transfected protoplasts, we found that GUS activities increased ∼5-folds in the presence of IAA (**Figure 1B**). We then generated *PRE6p:GUS* transgenic plants and assayed the auxin response of the reporter gene in plants. As shown in **Figure 1C**, GUS staining in the *PRE6p:GUS* transgenic seedlings was mainly observed in vascular system, and upon auxin treatment, GUS staining was enhanced in nearly all the tissues, indicating that the *PRE6* promoter is functional. By using the *PRE6p:GUS* transgenic plants, we found that *PRE6* was expressed in all the tissues and organs at seedling stage, but mainly expressed in the top of filament and style at maturity (Supplementary Figure S1).

### Both Overexpression and Loss-of-Function of *PRE6* in Arabidopsis Resulted in Auxin-Related Phenotypes

**FIGURE 2:**
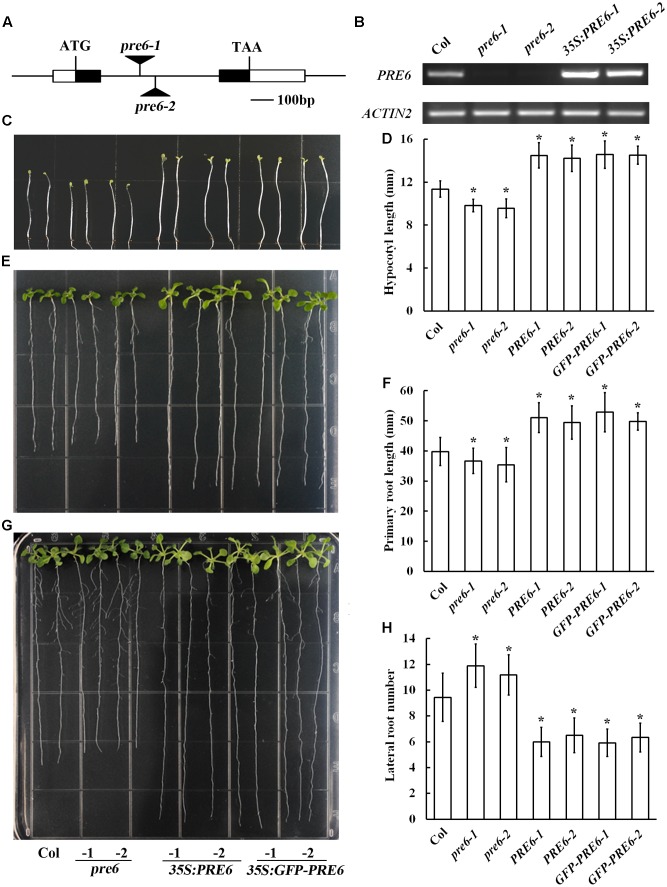
Phenotypes of the *pre6* mutants and the *PRE6* overexpression plants. **(A)** A diagram showing the T-DNA insertion sites in the *pre6-1* and *pre6-2* mutants. **(B)** Expression of *PRE6* transcript in the *pre6* mutants (*pre6-1* and *pre6-2*) and transgenic plants overexpressing *PRE6* (*35S:PRE6-1* and *35S:PRE6-2*). RNA was isolated from 14-day-old seedlings, and RT-PCR was used to examine the expression of *PRE6*. The expression of *ACT2* was used as a control. **(C)** Photographs of the 5-day-old etiolated seedlings of the Col wild type, the *pre6* mutants and the *35S:PRE6* transgenic plants. Sterilized seeds were grown on vertically placed 1/2 MS plates in darkness, and photographs were taken under a microscope. **(D)** Hypocotyl length of the 5-day-old etiolated seedlings of the Col wild type, the *pre6* mutants and the *35S:PRE6* transgenic plants. Photographs were taken under a microscope, and ImageJ software was used to measure the hypocotyl length. Data represent the mean ± SD of 33–35 seedlings. ^∗^Significantly different from the Col wild type seedlings (*p* < 0.01). **(E)** Photographs of the 10-day-old light-grown seedlings of the Col wild type, the *pre6* mutants and the *35S:PRE6* transgenic plants. Sterilized seeds were grown on vertically placed 1/2 MS plates, and photographs were taken under a microscope. **(F)** Primary root lengths of the 10-day-old light-grown seedlings of the Col wild type, the *pre6* mutants and the *35S:PRE6* transgenic plants. Photographs were taken under a microscope, and ImageJ software was used to measure the primary root length. Data represent the mean ± SD of 33 seedlings. ^∗^Significantly different from the Col wild type seedlings (*p* < 0.01). **(G)** Photographs of the 14-day-old light-grown seedlings of the Col wild type, the *pre6* mutants and the *35S:PRE6* transgenic plants. Sterilized seeds were grown on vertically placed 1/2 MS plates, and photographs were taken under a microscope. **(H)** Number of lateral roots in 14-day-old light-grown seedlings of the Col wild type, the *pre6* mutants and the *35S:PRE6* transgenic plants. Data represent the mean ± SD of at least 25 seedlings. ^∗^Significantly different from the Col wild type seedlings (*p* < 0.01).

Previous studies indicated that overexpression of *PRE6*, as well as *PRE1, PRE2, PRE3*, and *PRE4* increased hypocotyl length in the transgenic plants (Hyun and Lee, 2006; Lee et al., 2006; Mara et al., 2010; Hong et al., 2013; Gommers et al., 2017). To further examine whether Arabidopsis plants with altered expression level of *PRE6* may exhibit auxin-related phenotypes, we generated Arabidopsis transgenic plants overexpressing *PRE6*, isolated two independent T-DNA insertion alleles of *PRE6*, *pre6-1*, and *pre6-2* (**Figure 2A**), and compared their phenotypes by growing them side-by-side under same conditions. RT-PCR analysis showed that the *PRE6* transcript was elevated in the *35S:PRE6* transgenic plants, confirming their overexpression status, and that the full-length transcript of *PRE6* was undetectable in the *pre6-1* and *pre6-2* mutants, suggesting that they are likely representing loss-of-function alleles of *PRE6* (**Figure 2B**).

As expected, we found that dark-grown *35S:PRE6* transgenic seedlings had longer hypocotyls (**Figures 2C,D**). It has been reported recently that no difference on hypocotyl length is observed in light-grown *pre6* mutants and Col wild type seedlings (Gommers et al., 2017). However, we found that the hypocotyl of *pre6* mutants was slightly but statistically shorter than that of the Col wild type seedlings in dark-grown condition (**Figures 2C,D**). Similar to the results observed with hypocotyl length, we found that the primary roots were longer in the *35S:PRE6* transgenic seedlings, and shorter in the *pre6* mutants (**Figures 2E,F**). On the other hand, we found that *35S:PRE6* transgenic plants produced fewer, but *pre6* mutants produced more lateral roots than Col wild type seedlings (**Figures 2G,H**).

### *35S:PRE6* Transgenic Plants Are Hyposensitive Whereas *pre6* Mutants Are Hypersensitive to Auxin

Having shown that Arabidopsis plants with altered expression level of *PRE6* exhibited auxin-related phenotypes (**Figure 2**), we further examined the auxin response of the *35S:PRE6* transgenic plants and *pre6* mutants in terms of hypocotyl elongation, primary root elongation and lateral root formation.

**FIGURE 3:**
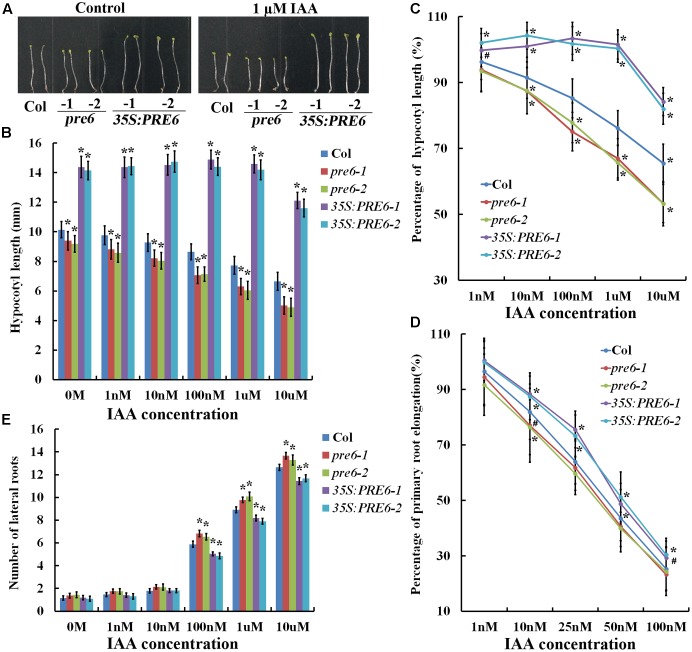
Auxin sensitivities of the *pre6* mutants and the *PRE6* overexpression plants. **(A)** Hypocotyls in the 5-day-old etiolated seedlings of the Col wild type, the *pre6* mutants and *PRE6* transgenic plants in the absence and presence of 1 μM IAA. Sterilized seeds were grown on vertically placed 1/2 MS plates with or without 1 μM IAA in darkness, and photographs were taken under a microscope. **(B)** Hypocotyl length of the 5-day-old etiolated seedlings of the Col wild type, the *pre6* mutants and *PRE6* transgenic plants in the absence and presence of IAA. Photographs were taken under a microscope, and ImageJ software was used to measure the hypocotyl length. Data represent the mean ± SD of 33–35 seedlings. ^∗^Significantly different from the Col wild type seedlings (*p* < 0.01). **(C)** Percentage of hypocotyl length. The percentages of hypocotyl length were calculated by comparing the hypocotyl length of the seedlings at the presence of IAA to those of the control seedlings. Data represent the mean ± SD of 33–35 seedlings. ^∗^*p* < 0.01, ^#^*p* < 0.05 significantly different from the Col wild type seedlings. **(D)** Percentage of primary root elongation. Five-day-old seedlings grown on a vertically placed 1/2 MS plates were transferred to new 1/2 MS plates supplemented with different concentrations of IAA, grown vertically for another 5 days, new elongated roots were measured, and the percentages of primary root elongation were calculated by comparing the primary root length of the seedlings at the presence of IAA to those of the control seedlings. Data represent the mean ± SD of 30 seedlings. ^∗^*p* < 0.01, ^#^*p* < 0.05 significantly different from the Col wild type seedlings. **(E)** Lateral root formation. Five-day-old seedlings grown on a vertically placed 1/2 MS plates were transferred to new 1/2 MS plates supplemented with different concentrations of IAA, and number of lateral roots was counted 5 days after the transfer. Data represent the mean ± SD of 25–30 seedlings. ^∗^Significantly different from the Col wild type seedlings (*p* < 0.05).

To examine hypocotyl elongation in response to auxin, seeds of Col wild type, *pre6* mutants and *35S:PRE6* transgenic plants were sown directly on 1/2 MS plates containing different concentrations of IAA, and hypocotyl length was measured 5 days after the plates were kept in darkness. As shown in **Figures 3A,B**, auxin at all the concentrations tested inhibited hypocotyl elongation in both the Col wild type and the *pre6* mutants, but showed little, if any effects in the *35S:PRE6* transgenic plants at lower concentrations (less than 1 μM). Analysis of percentage of hypocotyl elongation indicated that the *pre6* mutants are more sensitive to auxin than the Col wild type plants (**Figure 3C**).

To examine the effects of auxin on primary root elongation and lateral root formation, 5-day-old seedlings grown on vertically placed 1/2 MS plates were transferred to new plates containing different concentrations of IAA. Primary root elongation was measured, and number of lateral roots was counted 5 days after the transfer. We found that IAA at 1 nM has nearly no effects in the *35S:PRE6* transgenic plants, but inhibited primary root elongation in the Col wild type and *pre6* mutants (**Figure 3D**). IAA at 10 nM and higher, however, inhibited primary root elongation in all the plants examined (**Figure 3D**). On the other hand, we found that the *35S:PRE6* transgenic plants produced less, whereas the *pre6* mutants produced more lateral roots when compared with the Col wild type plants (**Figure 3E**). Taken together, these results suggest that the *35S:PRE6* transgenic plants were hyposensitive whereas *pre6* mutants were hypersensitive to auxin, indicating that PRE6 may negatively regulate auxin signaling in Arabidopsis.

### PRE6 Is a Transcriptional Repressor

PRE proteins are atypical bHLH transcription factors that lack DNA binding basic motif in the bHLH domain, thus it was proposed that PRE proteins act as negative regulator of bHLH transcription factors via the formation of heterodimers (Hyun and Lee, 2006). Consistent with this, it has been shown that co-expression of *PRE6* significantly reduced the transcriptional activation activities of HFR1 (Hong et al., 2013). Our results described above indicate that PRE6 is a negative regulator of auxin signaling in Arabidopsis. To further examine how PRE6 may regulate auxin signaling, we decided to examine whether PRE6 may regulate auxin response gene expression in Arabidopsis.

**FIGURE 4:**
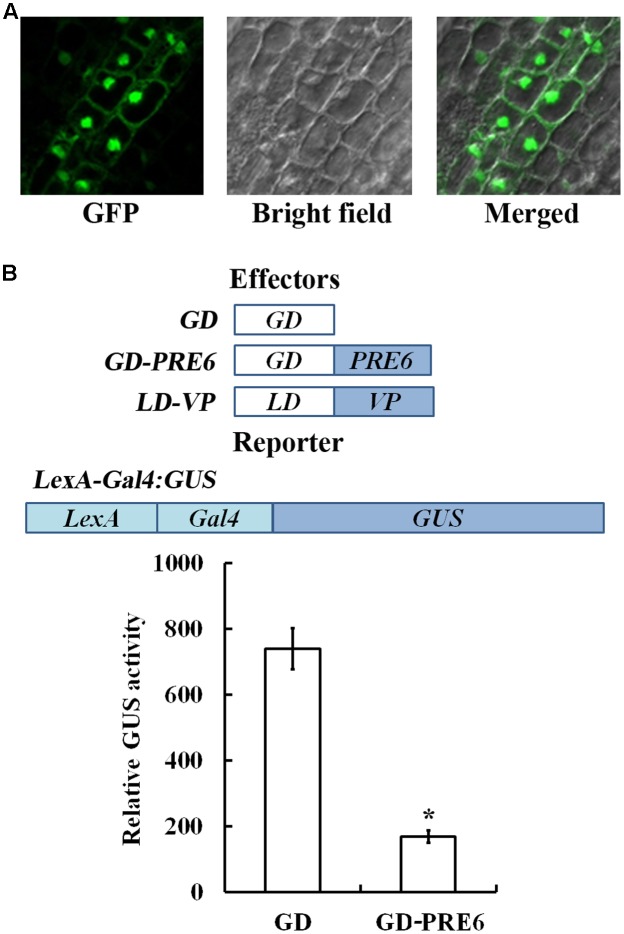
PRE6 is a transcriptional repressor. **(A)** Subcellular localization of PRE6. GFP fluorescence in the root tips of 7-day-old *35S:GFP-PRE6* transgenic seedlings were observed and photographed under a fluorescence microscopy. Left, GFP channel; middle, bright field; right, merged image. **(B)** PRE6 is a transcriptional repressor. Plasmids of the effector *LD-VP* and *GD-PRE6* or *GD* alone as a control, and the reporter *LexA:Gal4:GUS* (diagrammed on the top) plasmids were co-transfected into protoplasts isolated from rosette leaves of 3 to 4-week-old Col wild type plants, the protoplasts were incubated for 20–22 h at room temperature in darkness, and GUS activity was measured. Data represent the mean ± SD of three replicates. ^∗^Significantly different from the GD control (*p* < 0.01).

Previous results have only showed that PRE6 may not function as a transcriptional activator (Hong et al., 2013), thus we wanted to examine if PRE6 may function as a transcriptional repressor. Because previous protoplast transfection assays with *PRE6-GFP* plasmids indicated that PRE6 is localized in both nucleus and cytosol (Hong et al., 2013), we first wanted to examine if this is the case by generating transgenic plants expressing *GFP-PRE6*. As shown in **Figure 2**, the *35S:GFP-PRE6* transgenic seedlings showed a phenotype similar to that of the *35S:PRE6* transgenic seedlings, suggesting that GFP-PRE6 fusion proteins are functional. By examining GFP florescence in the roots of the *35S:GFP-PRE6* transgenic seedlings, we found that PRE6 is predominantly localized in the nucleus, but may also associate with membranes (**Figure 4A**). However, we could not rule out the possibility that the proteins were present in cytoplasm, but pushed to the cell periphery by the vacuole, which makes it looks like they were associated with membranes. We then examined the transcriptional activities of PRE6 by using Arabidopsis mesophyll protoplast transfection system. In this system, the transactivator LD-VP is recruited to the *LexA* promoter, whereas GD-PRE6 is recruited to *Gal4* promoter of *LexA-Gal4:GUS* reporter gene. As shown in **Figure 4B**, co-transfection of the plasmids of the effector gene *GD*, *LD-VP* and the reporter gene *LexA-Gal4:GUS* resulted in the activation of the reporter gene, whereas co-transfection of the effector gene *GD-PRE6*, *LD-VP* and the reporter gene *LaxA-Gal4:GUS* resulted in the repression of the reporter gene activated by LD-VP, suggesting that PRE6 is a transcriptional repressor.

### PRE6 Regulates Auxin Response Gene Expression

**FIGURE 5:**
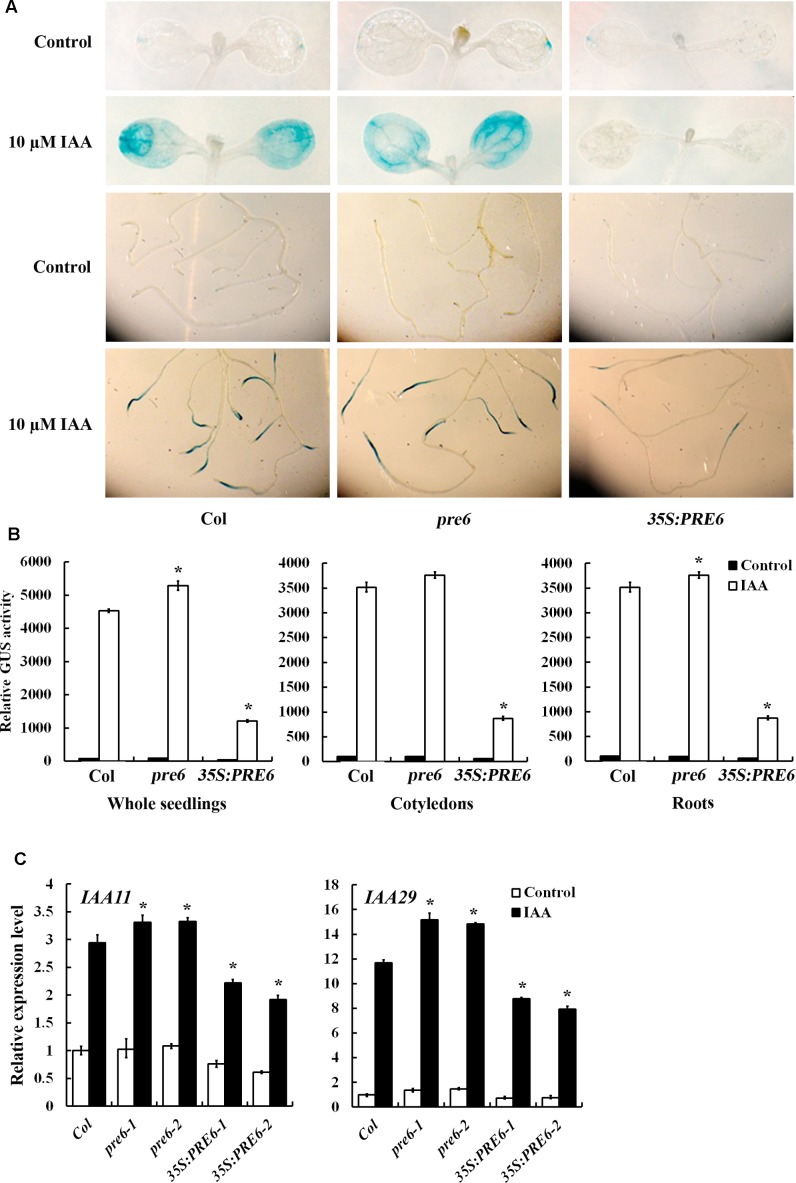
Expression of *DR5: GUS* reporter and endogenous Aux/IAA genes in the *pre6* mutants and the *PRE6* overexpression plants. **(A)** Expression of *DR5:GUS* reporter gene in the *pre6* mutants and the *PRE6* overexpression plants in response to exogenously applied IAA. Five-day-old seedlings with integrated *DR5:GUS* reporter gene were treated with 10 μM IAA for 12 h, then GUS activity was stained by using *X*-Gluc. Photographs were taken under a dissecting microscopy. **(B)** Quantitative analysis of GUS activities in the seedlings. After treated with 10 μM IAA for 12 h, seedlings were frozen in liquid nitrogen, proteins were extracted and GUS activity was measured. Data represent the mean ± SD of three replicates. ^∗^Significantly different from that of the control (*p* < 0.05). **(C)** Expression of *IAA11* and *IAA29* in response to exogenously applied IAA. Fourteen-day-old seedlings were treated with 10 μM IAA for 4 h, then total RNA was isolated and subjected to quantitative RT-PCR analysis. *ACT2* was used as a reference gene, and expression of each *IAA11* and *IAA29* in Col wild type seedlings in the absence of IAA was set as 1. Data represent the mean ± SD of three replicates. ^∗^Significantly different from that of the control (*p* < 0.05).

The results described above demonstrated that PRE6 is a transcriptional repressor and it is involved in the regulation of auxin response (**Figures 3**, **4**). To determine how PRE6 may regulate auxin response in Arabidopsis, we examined the effects of PRE6 on the expression of auxin response genes. The transgenic plants with the integrated *DR5:GUS* auxin response reporter gene were crossed with *35S:PRE6* transgenic plants and the *pre6* mutants, and the expression of the reporter genes in the absence and presence of auxin was examined by GUS staining. As shown in **Figure 5A**, in the absence of auxin, the *GUS* expression pattern in the *35S:PRE6* transgenic plants and the *pre6* mutants was similar to that of the Col wild type. However, in the presence of auxin, *GUS* expression was dramatically increased in the Col wild type and the *pre6* mutant seedlings, but not in the *35S:PRE6* transgenic seedlings (**Figure 5A**). Quantitative analysis showed that, in the presence of auxin, the *GUS* activity increased slightly in *35S:PRE6* transgenic seedlings, but decreased ∼4-folds as compared to the Col wild type and the *pre6* mutant seedlings (**Figure 5B**).

We also examined the expression of endogenous *Aux/IAA* genes in response to exogenously applied IAA by using qRT-PCR. We found that the expression levels of *IAA11* and *IAA29* in response to IAA were increased in the *pre6* mutants, but decreased in the *35S:PRE6* transgenic seedlings when compared to the Col wild type seedlings (**Figure 5C**).

### ARF5 and ARF8 Directly Regulate the Expression of *PRE6*

Auxin response genes are activated by ARF activators including ARF5, ARF6, ARF7, ARF8, and ARF19 (Wang et al., 2005). Because the expression of *PRE6* was induced by auxin (**Figure 1**), we examined if any of the ARF activator may regulate the expression of *PRE6* by using the Arabidopsis protoplast transient transfection assay. In this assay, plasmids of the ARF activator genes and the *PRE6p:GUS* reporter gene were co-transfected into the protoplasts, and GUS activity was measured after the transfected protoplasts were incubated overnight.

**FIGURE 6:**
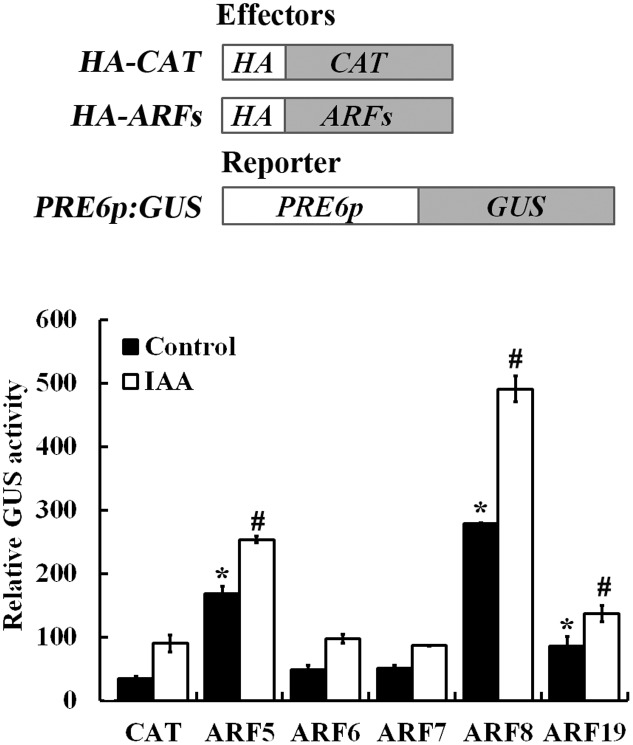
ARF5 and ARF8 activate *PRE6* expression in transfected protoplasts. Plasmids of the ARF effectors and the *PRE6p:GUS* reporter (diagrammed on the top) were co-transfected into protoplasts isolated from rosette leaves of 3 to 4-week-old Col wild type plants, the protoplasts were incubated for 20–22 h at room temperature in darkness, and GUS activity was measured. Co-transfection of CAT (chloramphenicol acetyltransferase) was used as a control. Data represent the mean ± SD of three replicates. ^∗^Significantly different from the CAT control in the absence of IAA (*p* < 0.05), ^#^Significantly different from the CAT control in the presence of IAA (*p* < 0.05).

As shown in **Figure 6**, in the absence of auxin, ∼4 and ∼6-folds increased expression of the *GUS* gene was obtained when the reporter was co-transfected with *ARF5* and *ARF8*, respectively, and GUS activity was further increased in the presence of auxin. The results also showed that co-transfection of *ARF19* slightly induced the reporter gene expression, whereas co-transfection of *ARF6* and ARF7 has little, if any effects on the expression of the reporter gene (**Figure 6**).

**FIGURE 7:**
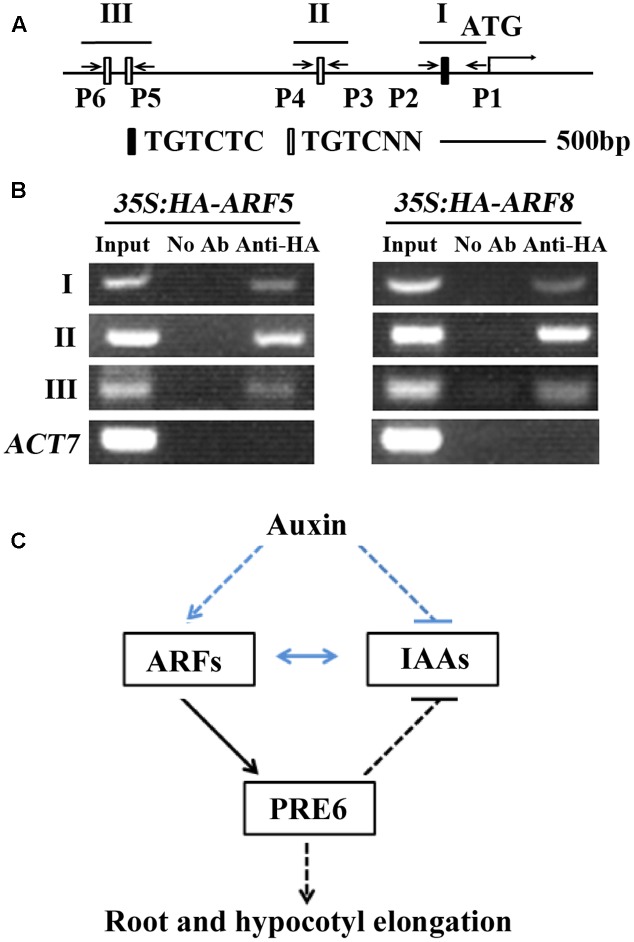
ARF5 and ARF8 are associated with the promoter region of *PRE6*. **(A)** ARF-binding sites in the 2 kb sequence upstream of the start codon of *PRE6*. I, II, and III indicate the regions for PCR amplification; P1 to P6 indicate the primers for PCR reactions. **(B)** Chromatin immunoprecipitation (ChIP) assay in transfected protoplasts. Plasmids of *HA-ARF5* or *HA-ARF8* were transfected into protoplasts isolated from rosette leaves of 3 to 4-week-old Col wild type plants, the transfected protoplasts were incubated for 20–22 h at room temperature in darkness, and ChIP assays were performed using anti-HA antibodies. Rabbit pre-immune serum was used as a mock control. Primer sets spanning the auxin response elements were used in PCR reactions. Amplification of *ACT7* was used as a control. **(C)** A model illustrating the role of PRE6 in plant growth and development and auxin singling. ARFs directly activate the expression of *PRE6*, PRE6 regulates root and hypocotyls elongation, and it also serve as a feedback regulation loop in the regulation of auxin signaling via inhibiting the expression of Aux/IAA genes. Lines in blue indicate previously known results, and lines in black indicate results reported in this research.

It is well-known that ARFs regulate auxin response gene expression via binding to the TGTCTC auxin response elements (Ulmasov et al., 1995, 1997a,b, 1999). Sequence analysis showed that *PRE6* has one canonical TGTCTC element and three TGTC core elements within the 2 kb region upstream of its start codon (**Figure 7A**), indicating that ARF5 and ARF8 may regulate *PRE6* expression via binding to its promoter. To test if this is the case, we performed ChIP assay to determine the association of ARF5 and ARF8 proteins to the promoter. Plasmids of *ARF5* and *ARF8* (with an HA tag) were transfected into Arabidopsis protoplasts, and anti-HA antibodies were used for immunoprecipitation. Immunoprecipitated DNA was amplified by using PCR with primers spanning the canonical TGTCTC element and the TGTC core elements (**Figure 7A**). Specific PCR products of the expected size were obtained for the three regions, but no bands were obtained when rabbit pre-immune serum was used for immunoprecipitation (**Figure 7B**). These results suggest that *PRE6* is a target gene of ARF5 and ARF8.

## Discussion

PREs have been reported to be involved in the regulation of gibberellin, brassinosteroid, temperature and light signaling in Arabidopsis (Lee et al., 2006; Wang et al., 2009; Zhang et al., 2009; Mara et al., 2010; Bai et al., 2012; Castelain et al., 2012; Oh et al., 2014). It has also been suggested that PRE1 is involved in the cross-talking between several different plant hormones including gibberellin, brassinosteroid and auxin (Oh et al., 2014), and the expression of *PRE1* has been shown to be induced by several different plant hormones including gibberellin, auxin, and brassinosteroid (Lee et al., 2006; Zhang et al., 2009). In this study, we found that *PRE6* is an auxin response gene, and it regulates auxin response in Arabidopsis.

### *PRE6* Is a Direct Target of ARF5 and ARF8

Among the six *PRE* genes, *PRE3* was identified a target of ARF5 (Schlereth et al., 2010), and the expression of *PRE1* and *PRE5* has been shown to be induced by auxin (Zhang et al., 2009; Oh et al., 2014). Our qRT-PCR results showed that, in addition to *PRE1*, the expression of three other *PRE* genes, i.e., *PRE2*, *PRE5*, and *PRE6* were also induced by auxin, with *PRE6* being the highest responsive *PRE* gene to auxin (**Figure 1A**). Our results showed that the expression level of *PRE1* increased only about twofold in response to auxin (**Figure 1A**), a result similar to the previous observation (Zhang et al., 2009). Unexpectedly, our results showed that the expression of *PRE3* was slightly down regulated by auxin (**Figure 1A**), although *PRE3* has been shown to be a target of ARF5 (Schlereth et al., 2010).

Auxin response of *PRE6* was further confirmed by using *PRE6p:GUS* reporter gene in transfected protoplasts (**Figure 1B**), as well as in stable transformed plants (**Figure 1C**). The expression of auxin responsive genes are regulated by the interplay of ARFs activator and Aux/IAA proteins (Ulmasov et al., 1997a, 1999; Tiwari et al., 2003, 2004; Wang et al., 2005; Guilfoyle and Hagen, 2007; Chandler, 2016). By using protoplast transfection assays, we found that the expression of *PRE6* was activated by ARF5 and ARF8, but not by AFR6 and ARF7, whereas ARF19 also slightly activated the expression of *PRE6* (**Figure 6**). Sequencing analysis showed that there are four canonical or core auxin response elements in the promoter region of *PRE6*, and ChIP assay indicated that both ARF5 and ARF8 can be associated with those elements (**Figure 7**), indicating that *PRE6* is a direct target of ARF5 and ARF8.

### PRE6 Is Involved in the Regulation of Auxin Signaling in Arabidopsis

Alteration on hypocotyl and primary root length and lateral root numbers are observed in some well characterized auxin mutants such as *iaa7*, *tir1-1*, and *iaa14* (Fukaki et al., 2002; Nakamura et al., 2006; Strader et al., 2008), and are considered as auxin-related phenotypes (Chapman and Estelle, 2009; Weijers and Wagner, 2016). Both the *pre6* mutants and *35S:PRE6* transgenic seedlings showed auxin related phenotypes (**Figure 2**). Auxin response analysis showed that *pre6* mutants are hypersensitive, whereas *35S:PRE6* transgenic plants are hyposensitive to auxin (**Figure 3**), indicating that PRE6 is a negative regulator of auxin signaling. Indeed, we found that the expression of the *DR5:GUS* reporter, as well as some of the endogenous *Aux/IAA* genes including *IAA11* and *IAA29* in response to exogenously applied IAA was reduced in the *35S:PRE6* transgenic plants (**Figure 5**). Consistent with this observation, transfection assays in protoplasts indicated that PRE6 function as a transcriptional repressor (**Figure 4**). However, considering that, expression of *IAA11* and *IAA29* was still highly induced by exogenously applied IAA (**Figure 5**), it is unlikely that PRE6 may directly regulate the expression of *IAA11* and *IAA29*. It may be of great interest to find out how PRE6 may regulate the expression of *Aux/IAA* genes. Considering that stabilities of Aux/IAA proteins play an important role in auxin signaling, and PRE6 also function as a negative regulator of auxin signaling, it may also be of interest to examine whether PRE6 can be ubiquitinated like Aux/IAA proteins for degradation.

Auxin signaling is mainly controlled by the interplay of ARF activators and Aux/IAA protein. Our results showed that *PRE6* is a direct target of ARF5 and ARF8 (**Figures 6**, **7**), indicating that PRE6 functions downstream of ARF5 and ARF8 to regulate auxin signaling. Consistent with this, it has been reported that dark-grown *arf6-2*, *arf8-3*, and *arf6-2 arf8-3* seedlings produced short hypocotyl (Nagpal et al., 2005; Oh et al., 2014), a phenotype similar to that of *pre6* mutant seedlings (**Figure 2**). It should be noted that no difference in hypocotyl length was observed in light-grown *pre6* mutants and Col wild type seedlings (Gommers et al., 2017). It is likely because light may affect auxin signaling, thus affecting plant growth and development.

PRE6 has been shown to regulate photomorphogenesis and light signaling via interacting with HFR1 (LONG HYPOCOTYL IN FAR-RED), a bHLH protein known to regulate photomorphogenesis in Arabidopsis, to interfere the interaction between HFR and PIF4 (PHYTOCHROMEINTERACTING FACTOR4), another bHLH protein known to regulate photomorphogenesis (Hyun and Lee, 2006; Hong et al., 2013). PIF4 has also been shown to interact with ARF6 to regulate hypocotyl cell elongation (Oh et al., 2014), thus it is likely that PRE6 may play a negative feedback role in auxin signaling by interfering the interaction of ARF activators and bHLH transcription factors, thus affecting the function of ARF activators. However, because PRE6 functioned as a transcriptional repressor in our protoplast transfection assays (**Figure 4**), and it suppressed the expression of the *DR5:GUS* reporter gene and some *Aux/IAA* genes in the transgenic plants, we could not rule out the possibility that PRE6 may be directly involved in the regulation of the expression of the auxin responsive genes.

PREs have been shown to function redundantly to regulate plant growth and development, as well as plant response to hormone such as gibberellins and brassinosteroid and light signaling (Hyun and Lee, 2006; Lee et al., 2006; Bai et al., 2012; Oh et al., 2014). PRE1 has been shown to cooperate with ARF6 to regulate hypocotyl cell elongation in Arabidopsis (Oh et al., 2014), and *PRE3* has been shown to be a target of ARF5 (Schlereth et al., 2010). Considering that expression of *PRE2* and *PRE5* were also induced by exogenously applied IAA (**Figure 1A**), and PREs share high identities and similarities at the amino acid level (Mara et al., 2010), it is very likely that PREs may function redundantly to regulate auxin signaling in Arabidopsis.

We found in this study that *PRE6* is an auxin response gene whose expression is regulated by ARF5 and ARF8, that PRE6 is a transcriptional repressor, and that PRE6 negatively regulates auxin signaling in Arabidopsis. Because Aux/IAA proteins can dimerize with ARFs to repress their functions, and inhibition of Aux/IAA genes by PRE6 may relieve this repression, it is very likely that ARF5 and ARF8 activated expression of *PRE6* may function as positive feedback in auxin signaling (**Figure 7C**). Taken together, these results provide new insights into the function of PREs, and the regulation of auxin signaling in Arabidopsis.

## Author Contributions

SW and J-GC conceived the study and designed the experiments. KZ, YW, NZ, QJ, XW, and CH performed the experiments. KZ and SW analyzed the data. KZ and SW drafted the manuscript. All the authors participated in the revision of the manuscript.

## Conflict of Interest Statement

The authors declare that the research was conducted in the absence of any commercial or financial relationships that could be construed as a potential conflict of interest. The reviewer DS and handling Editor declared their shared affiliation.

## Footnotes

1 https://www.graphpad.com/quickcalcs/ttest1/?Format=SD
