# Supplementary figures and images for: Involvement of PACLOBUTRAZOL RESISTANCE6/KIDARI, an Atypical bHLH Transcription Factor, in Auxin Responses in Arabidopsis
Source: Front Plant Sci. 2017 Oct 24;8:1813. doi: 10.3389/fpls.2017.01813 (PMC5660721; doi:10.3389/fpls.2017.01813)

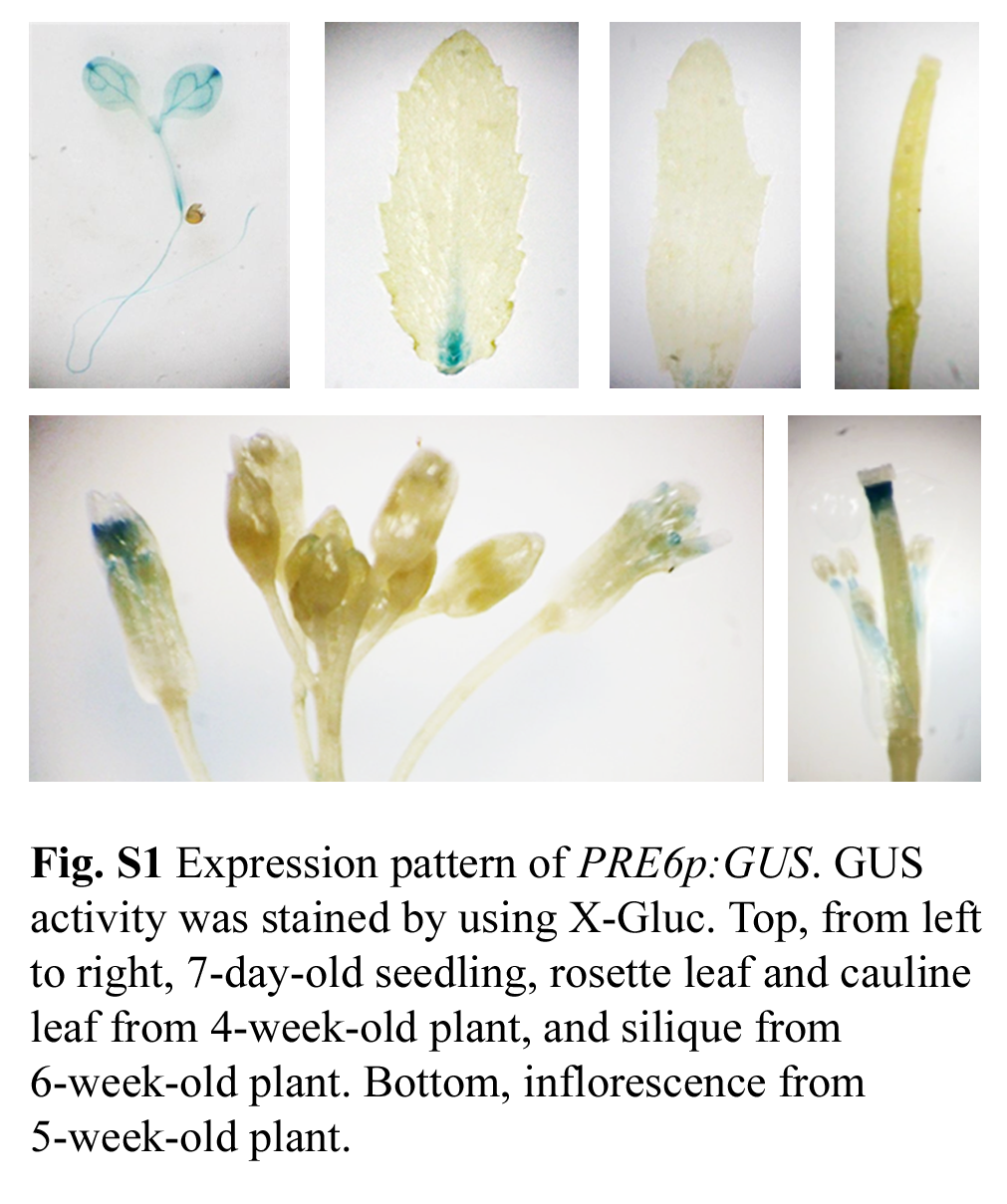

Supplement: Supplementary file 1 [file Image_1.TIF]
